# Mechanotransduction Activity Facilitates Hair Cell Toxicity Caused by the Heavy Metal Cadmium

**DOI:** 10.3389/fncel.2020.00037

**Published:** 2020-02-20

**Authors:** Caleigh Schmid, Isabella Alampi, Jay Briggs, Kelly Tarcza, Tamara M. Stawicki

**Affiliations:** ^1^Program in Neuroscience, Lafayette College, Easton, PA, United States; ^2^Department of Biological Structure, University of Washington, Seattle, WA, United States

**Keywords:** cadmium, hair cells, heavy metals, ototoxicity, zebrafish

## Abstract

Hair cells are sensitive to many insults including environmental toxins such as heavy metals. We show here that cadmium can consistently kill hair cells of the zebrafish lateral line. Disrupting hair cell mechanotransduction genetically or pharmacologically significantly reduces the amount of hair cell death seen in response to cadmium, suggesting a role for mechanotransduction in this cell death process, possibly as a means for cadmium uptake into the cells. Likewise, when looking at multiple cilia-associated gene mutants that have previously been shown to be resistant to aminoglycoside-induced hair cell death, resistance to cadmium-induced hair cell death is only seen in those with mechanotransduction defects. In contrast to what was seen with mechanotransduction, significant protection was not consistently seen from other ions previously shown to compete for cadmium uptake into cells or tissue including zinc and copper. These results show that functional mechanotransduction activity is playing a significant role in cadmium-induced hair cell death.

## Introduction

Hearing loss is one of the most common sensory disorders affecting upwards of 20% of Americans over the age of 12 (Lin et al., 2011; Goman and Lin, 2016). One common cause of hearing loss is the death of sensory hair cells. This is also a cause of vestibular dysfunction. Hair cells are sensitive to numerous insults including loud noises, certain therapeutic drugs, and aging (Cheng et al., 2005; Schacht et al., 2012; Yamasoba et al., 2013; Kurabi et al., 2017). There is also evidence that environmental toxins such as heavy metals can lead to hearing loss (Choi and Kim, 2014; Schaal et al., 2017), though this form of hearing loss has not been as well-established or studied as other forms.

Heavy metal toxicity, in general, is a growing environmental concern. Heavy metals are naturally occurring and while some are required in the body at trace levels, higher levels are often associated with toxicity. Other heavy metals, such as cadmium, have no known function or benefit to animals. Cadmium is used in batteries and some pigments and is produced as a by-product of zinc mining. It can be released into the environment through industrial run-off from a variety of different sources including mine drainage water, sewage treatment plans, and hazardous waste sites (IARC, 2012). Working in certain industries comes with a risk of occupational exposure to cadmium, and nonoccupational exposures can occur through diet or smoking (Faroon et al., 2012). Elevated levels of blood or urinary cadmium correlate with a number of health problems including dysfunction of the kidneys, liver, cardiovascular system, osteoporosis, and cancer (Gallagher et al., 2008; Ferraro et al., 2010; Tellez-Plaza et al., 2012; Hyder et al., 2013; García-Esquinas et al., 2014).

Studies looking at the link between cadmium exposure and hearing loss have had conflicting results. Some studies have shown increased hearing thresholds or balance impairments in individuals with higher blood cadmium levels (Choi et al., 2012; Min et al., 2012; Choi and Park, 2017). However, other studies have failed to show a significant association between elevated urinary or blood cadmium levels and hearing loss (Shiue, 2013; Kang et al., 2018; Liu et al., 2018). Some of these conflicting results may come from the different methods of quantifying cadmium levels and hearing loss used by the different groups. Studies in rodents have likewise drawn varying conclusions over whether or not cadmium exposure can cause hearing loss. Hair cell death in organ of Corti cultures and changes in auditory brain response (ABR) and distortion product otoacoustic emissions (DPOAEs) have been seen in mice and rats by some groups (Ozcaglar et al., 2001; Kim et al., 2008; Liu et al., 2014). However, other groups have failed to see vestibular dysfunction or hearing loss following exposure to cadmium alone (Whitworth et al., 1999; Klimpel et al., 2017; Carlson et al., 2018). Again different groups have used different cadmium treatment paradigms which may be responsible for the conflicting results. Defects in auditory and lateral line systems have been more consistently seen in fish following cadmium exposure (Baker and Montgomery, 2001; Faucher et al., 2006, 2008; Low and Higgs, 2015; Sonnack et al., 2015; Montalbano et al., 2018), making fish a useful model to study the mechanisms by which cadmium may cause hair cell death. The lateral line is a superficial sensory structure in aquatic animals that is used to detect water movements and contains hair cells similar to those used for hearing and balance in the mammalian inner ear (Larsson, 2012). It has previously been shown that hair cells of the zebrafish lateral line system are sensitive to many of the same insults as mammalian hair cells (Harris et al., 2003; Ton and Parng, 2005; Ou et al., 2007), and the presence of lateral line hair cells on the surface of the animal facilitates both observation of hair cells and the access of potential ototoxic drugs.

One open question regarding cadmium-induced hair cell death is how cadmium enters hair cells. Cells do not have designated cadmium channels or transporters as cadmium has no function in the cell. Instead, studies looking at other cell types have found that cadmium enters through channels and transporters for other ions. Studies have shown that cadmium can enter cells *via* zinc transporters ZIP8 and ZIP14, the iron transporter DMT1, the organic cation channels OCT1 and OCT2, and the TRP channels TRPV5, TRPV6, and TRPM7 (Olivi et al., 2001; Bannon et al., 2003; Dalton et al., 2005; Girijashanker et al., 2008; Lévesque et al., 2008; Fujishiro et al., 2009; Martineau et al., 2010; Kovacs et al., 2011, 2013; Soodvilai et al., 2011). It has also been shown that some metals such as copper and zinc can compete with cadmium for uptake into tissues, presumably due to the use of shared uptake routes (Barbier et al., 2004; Komjarova and Bury, 2014). Another potential uptake route for cadmium into hair cells is the hair cell mechanotransduction channel. Other hair cell toxicants including aminoglycoside antibiotics have been shown to require functional mechanotransduction to enter hair cells (Marcotti et al., 2005; Alharazneh et al., 2011). Cisplatin hair cell toxicity is also dependent on functional mechanotransduction activity (Thomas et al., 2013; Stawicki et al., 2014) though it remains unclear whether this is due to cisplatin entering through the mechanotransduction channel or mechanotransduction activity indirectly affecting toxicity (Vargo et al., 2017).

We have found that cadmium can consistently kill hair cells of the zebrafish lateral line system in a dose-dependent manner. This hair cell death is reduced following both genetic and pharmacological inhibition of hair cell mechanotransduction suggesting that mechanotransduction plays a role in cadmium-induced hair cell death, potentially as the route through which cadmium enters hair cells. In contrast to this, we did not see consistent significant protection from cadmium-induced hair cell death when cotreating fish with either zinc or copper, suggesting that these ions are not competing with cadmium for hair cell entry.

## Materials and Methods

### Animals

All experiments used 5-day post-fertilization (dpf) *Danio rerio* (zebrafish) larvae. Experiments were carried out with either *AB wild type zebrafish, *cdh23*^tj264^ (Nicolson et al., 1998; Söllner et al., 2004), *ift88*^tz288^ (Brand et al., 1996; Tsujikawa and Malicki, 2004), or *cc2d2a*^w38^ (Owens et al., 2008) mutants. Mutant alleles were maintained in the *AB background and experiments were carried out on offspring of incrosses of heterozygous parents comparing homozygous mutants to both homozygous and heterozygous wild-type siblings from the same clutch. Mutants were separated based on secondary phenotypes; vestibular defects in the case of *cdh23* mutants and body morphology defects in the case of *ift88* and *cc2d2a* mutants.

Larvae were raised in Petri dishes containing embryo media (EM) consisting of 1 mM MgSO_4_, 150 μM KH_2_PO_4_, 42 μM Na_2_HPO_4_, 1 mM CaCl_2_, 500 μM KCl, 15 mM NaCl, and 714 μM NaHCO_3._ They were housed in an incubator maintained at 28.5°C. The Lafayette College or the University of Washington Institution Animal Care and Use Committee approved all experiments.

### Drug Treatment

Fish were treated with cadmium chloride hemipentahydrate (Thermo Fisher Scientific, Waltham, MA, USA) dissolved in EM for 3 h at 28.5°C. Some fish were additionally treated with benzamil hydrochloride hydrate (Sigma-Adrich, St. Louis, MO, USA), zinc sulfate heptahydrate (Sigma-Adrich, St. Louis, MO, USA), or copper sulfate pentahydrate (Sigma-Adrich, St. Louis, MO, USA). A stock of benzamil was made at a concentration of 40 mM in dimethyl sulfoxide (DMSO, Sigma-Adrich, St. Louis, MO, USA). Control animals for the benzamil experiment were therefore treated with DMSO diluted in EM at a 1:200 ratio to match the DMSO levels in the benzamil group. Stocks of all other compounds were made in water and DMSO was not used for those experiments. For all treatments, fish were put into six well plates containing net-well inserts and the net-wells were moved to plates containing the different solutions the fish were exposed to. Following treatment, animals were washed three times in EM, euthanized with MS-222 and immediately fixed for immunostaining.

### Immunostaining and Hair Cell Counts

Fish used for immunohistochemistry were fixed for either 2 h at room temperature or overnight at 4°C in 4% paraformaldehyde. Antibody labeling was carried out as previously described (Stawicki et al., 2014). Fish used for hair cell counts were labeled with a rabbit anti-parvalbumin primary antibody (Thermo Fisher Scientific, Waltham, MA, USA, PA1-933) diluted at 1:1,000 in antibody block (5% goat serum in PBS, 0.2% Triton, 1% DMSO, and 0.2% BSA). Hair cells were counted by eye using either a Zeiss Axioplan 2 or an Accu-Scope EXC-350 under a 40× objective in the OP1, M2, IO4, O2, MI2, and MI1 neuromasts (Raible and Kruse, 2000) and then an average number of hair cells/neuromast was calculated for each animal. For representative neuromast images fish were imaged on an Accu-Scope EXC-350 using an Excelis MPX-5C pro camera and CaptaVision+ software.

Data is presented in graphs as the mean of the hair cell/neuromast number for all fish in a group ± standard deviation. In data presented as percentage of control, this hair cell/neuromast number for each fish was divided by the average hair cell/neuromast number of the appropriate control group and then converted to a percentage. Data was then presented in graphs as this normalized percent ± standard deviation of the normalized percent. We aimed to have ten animals per group for each experiment, however, in some cases, animals were lost during the staining process and therefore smaller sample sizes were used. The exact sample sizes for each experiment are listed in the figure legends. Statistics were calculated in GraphPad Prism 6 using either the average hair cell/neuromast number counted for each animal or the normalized percents for each animal.

## Results

### Acute Cadmium Treatment Can Kill Hair Cells of Larval Zebrafish in a Dose-Dependent Manner

**Figure 1:**
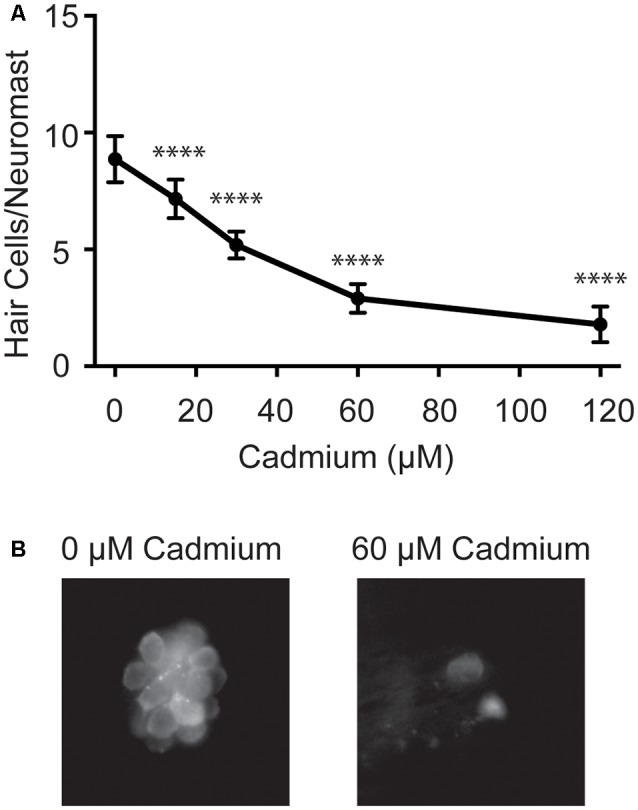
Cadmium kills hair cells in zebrafish larvae in a dose-dependent manner. **(A)** Treating 5-days post-fertilization (dpf) zebrafish with cadmium chloride doses ranging from 0 to 120 μM for 3 h showed an increasing amount of hair cell death with increasing cadmium chloride dose. Data are displayed as mean ± standard deviation. ****p < 0.0001 as compared to 0 μM cadmium chloride control by ANOVA and Dunnett’s *post hoc* test. Fish were fixed and stained with an anti-parvalbumin antibody and hair cells from six neuromasts from each fish were counted (OP1, M2, IO4, O2, MI2, and MI1) and averaged. *n* = 10 fish for the 0, 15, 60 and 120 μM cadmium chloride groups and *n* = 7 for the 30 μM cadmium chloride group. **(B)** Representative images of the MI1 neuromast following treatment with either 0 (left) or 60 (right) μM cadmium chloride.

To investigate potential mechanisms of cadmium uptake into hair cells we first worked to develop an acute cadmium treatment paradigm that would reliably kill hair cells. Cadmium has previously been shown to kill hair cells in both adult and larval zebrafish (Wang and Gallagher, 2013; Sonnack et al., 2015; Montalbano et al., 2018); however, none of these experiments quantified hair cell death in larval fish following acute treatment paradigms. We found that after a 3-h treatment with cadmium chloride, we could see a dose-dependent decrease in lateral line hair cell numbers (Figure 1). This treatment paradigm was thus used in subsequent experiments looking for protection from cadmium-induced hair cell death.

### Impaired Mechanotransduction Activity Reduces Hair Cell Death in Response to Cadmium

**Figure 2:**
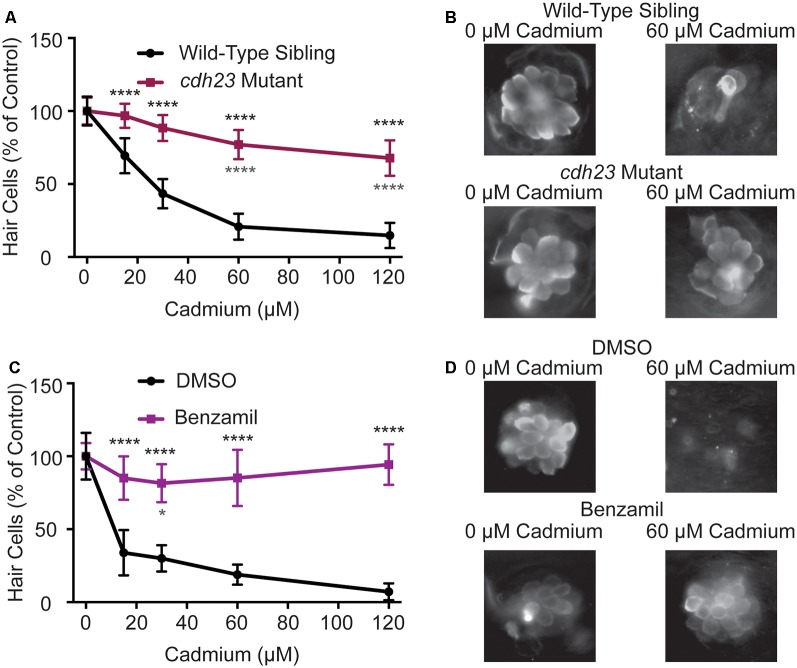
Cadmium toxicity is dependent on functional mechanotransduction activity. **(A)** *cdh2*3^*tj264*^ mutants, which lack functional mechanotransduction activity, are resistant to cadmium-induced hair cell death. **(B)** Representative images of the O2 Neuromast in wild-type siblings (top) and *cdh23* mutants (bottom) fish following treatment with either 0 (left) or 60 (right) μM cadmium chloride. **(C)** Treatment with 200 μM Benzamil, a mechanotransduction channel blocker, significantly blocks cadmium-induced hair cell death as compared to fish treated with the same amount of DMSO alone (a 1:200 dilution). **(D)** Representative images of the O2 Neuromast in DMSO-treated (top) and benzamil treated (bottom) fish following treatment with either 0 (left) or 60 (right) μM cadmium chloride. Due to reduced hair cell numbers in *cdh23* mutants data is normalized to the 0 cadmium chloride control for each treatment group. Data are displayed as mean ± standard deviation. *p < 0.05, ****p < 0.0001 by Two-Way ANOVA and Šídák multiple comparisons test. Black stars above the error bars denote significant differences in *cdh23* mutants or benzamil treated fish as compared to their respective wild-type sibling and DMSO control groups treated with the same dose of cadmium chloride. Gray stars below the error bars denote significant differences in *cdh23* mutants or benzamil treated fish at that cadmium chloride dose compared to the 0 cadmium chloride control group of the same mutant or drug condition. *n* = 10 fish for all groups.

Multiple hair cell toxicants have been shown to enter hair cells in a mechanotransduction-dependent manner (Marcotti et al., 2005; Alharazneh et al., 2011) and manipulations that decrease hair cell mechanotransduction activity frequently protect hair cells from toxicants (Seiler and Nicolson, 1999; Wang and Steyger, 2009; Thomas et al., 2013; Stawicki et al., 2014). To test if impairing mechanotransduction would likewise protect hair cells from cadmium-induced hair cell death, we both genetically and pharmacologically manipulated hair cell mechanotransduction activity in zebrafish larvae. First, we tested *cadherin 23* (*cdh2*3^tj264^) mutants, also known as *sputnik*. Cadherin 23 is one of the proteins that make up the tip links that link neighboring stereocilia in hair cells. Mutants no longer have tip links and thus do not have functional mechanotransduction activity (Nicolson et al., 1998; Söllner et al., 2004). We found that there was significantly less cadmium-induced hair cell death in these mutants at all doses tested though hair cell death was not eliminated at higher cadmium chloride doses (Figures 2A,B). We next pharmacologically inhibited mechanotransduction activity by cotreating fish with cadmium chloride and 200 μM benzamil, an analog of amiloride that has been shown to block hair cell mechanotransduction activity (Hailey et al., 2017). We again saw significant protection against cadmium-induced hair cell death at all cadmium chloride doses (Figures 2C,D).

**Figure 3:**
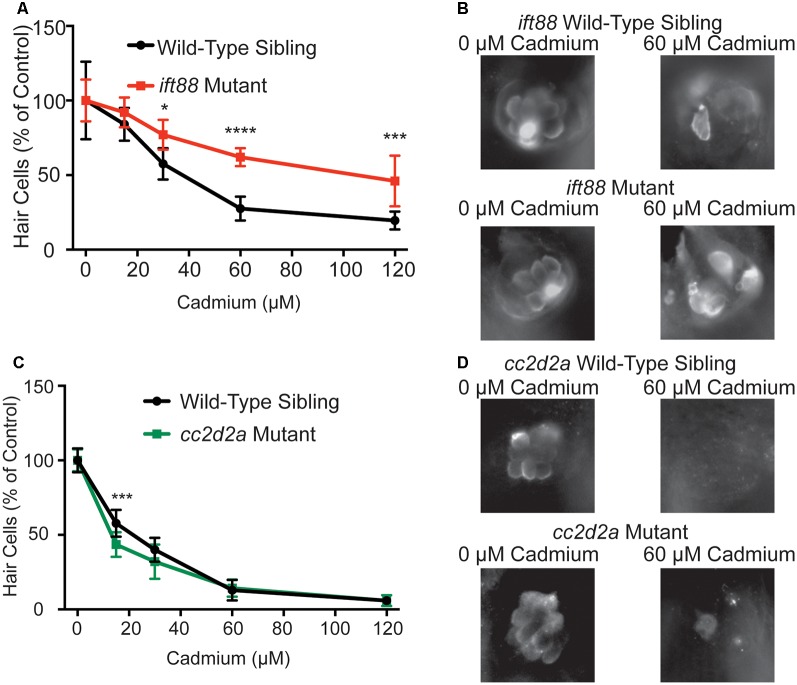
Genetic mutants resistant to neomycin-induced hair cell death due to impaired mechanotransduction activity are also resistant to cadmium-induced hair cell death. **(A)** *ift88*^tz288^ mutants, which have previously been shown to have reduced mechanotransduction activity (Kindt et al., 2012; Stawicki et al., 2016), are partially resistant to cadmium-induced hair cell death. **(B)** Representative images of the IO4 Neuromast in *ift88* wild-type siblings (top) and *ift88* mutant fish (bottom) following treatment with either 0 (left) or 60 (right) μM cadmium chloride. **(C)** *cc2d2a*^w38^ mutants, which have previously been shown to have normal mechanotransduction activity (Owens et al., 2008; Stawicki et al., 2016), are not resistant to cadmium-induced hair cell death. **(D)** Representative images of the IO4 Neuromast in *cc2d2a* wild-type siblings (top) and *cc2d2a* mutant fish (bottom) following treatment with either 0 (left) or 60 (right) μM cadmium chloride. Due to reduced hair cell number in *ift88* mutants data is normalized to the 0 cadmium chloride control for each treatment group. Data are displayed as mean ± standard deviation. *p < 0.05, ***p < 0.001, ****p < 0.0001 by Two-Way ANOVA and Šídák multiple comparisons test. For the *ift88* mutant experiment *n* = 10 fish for wild-type sibling 15 and 120 μM cadmium chloride and mutant 0, 15, and 30 μM cadmium chloride, *n* = 9 for wild-type sibling 0 μM cadmium chloride and *n* = 8 for wild-type sibling 30 and 60 μM cadmium chloride and mutant 60 and 120 μM cadmium chloride. For the *cc2d2a* experiment *n* = 10 fish for all groups.

We next tested whether genetic mutants identified through a screen looking for mutants resistant to aminoglycoside-induced hair cell death (Owens et al., 2008) would also be resistant to cadmium-induced hair cell death. We tested two different cilia-associated gene mutants, *ift88*^tz288^, and *cc2d2a*^w38^, both mutants contain premature stop codons in their respective genes (Tsujikawa and Malicki, 2004; Owens et al., 2008). The *ift88* mutants have previously been shown to have reduced responses to water jet stimulation (Kindt et al., 2012) and reductions in FM1-43 and neomycin uptake (Stawicki et al., 2016) suggesting that their resistance to neomycin-induced hair cell death is due to reduced neomycin uptake as a result of reduced mechanotransduction activity. In contrast to this *cc2d2a* mutants have been previously shown to have normal neomycin uptake and mechanotransduction activity (Owens et al., 2008; Stawicki et al., 2016). We found reduced hair cell death in response to cadmium chloride in *ift88* mutants (Figures 3A,B), further suggesting that mechanotransduction plays a role in cadmium-induced hair cell death. In contrast, we saw no reduction of hair cell death in *cc2d2a* mutants in response to cadmium chloride (Figures 3C,D).

### Zinc and Copper Cotreatment Do Not Protect Hair Cells From Cadmium-Induced Hair Cell Death as Effectively as Impairing Mechanotransduction Does

**Figure 4:**
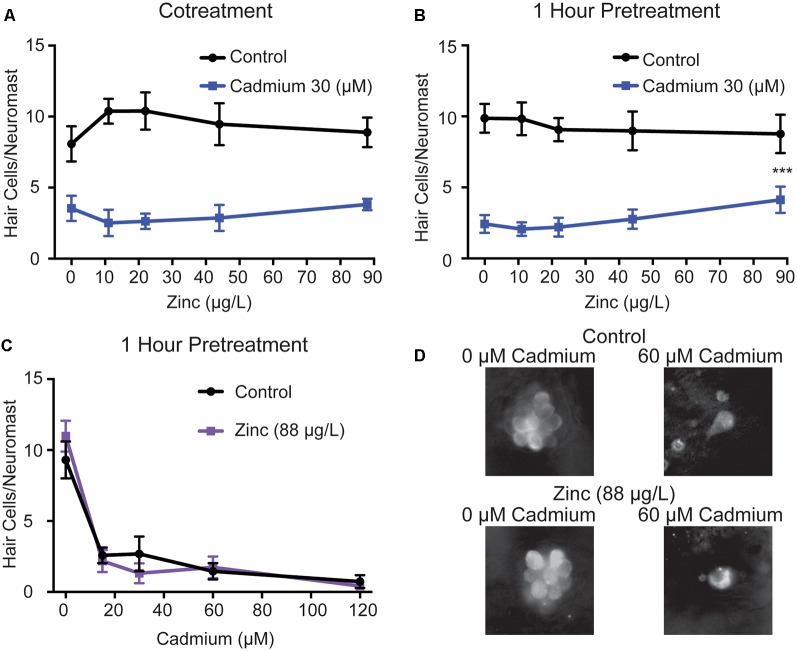
Zinc shows limited protection against cadmium-induced hair cell death. Zinc sulfate failed to reduce hair cell death when administered **(A)** alongside cadmium chloride and only showed a slight reduction at the highest dose tested when administered **(B)** 1 h before and during cadmium chloride treatment. This protection was not seen when tested against a range of cadmium chloride doses **(C)**. Data are displayed as mean ± standard deviation. ***p < 0.001 as compared to the 0 zinc sulfate control by Two-Way ANOVA and Šídák multiple comparisons test. *n* = 10 fish for all groups. **(D)** Representative images of the IO4 Neuromast in control (top) and zinc sulfate treated (bottom) fish following treatment with either 0 (left) or 60 (right) μM cadmium chloride.

As we still saw some hair cell death in *cdh23* mutants, which completely lack mechanotransduction, we wanted to test if there were alternative means by which cadmium could enter hair cells. Previous experiments have shown that cadmium enters other cell types through zinc transporters (Dalton et al., 2005; Fujishiro et al., 2009), and that zinc can block cadmium uptake (Barbier et al., 2004; Girijashanker et al., 2008). Zinc has also previously been shown to protect against impaired behavioral responses to odorants caused by cadmium in larval zebrafish (Heffern et al., 2018) and to protect against impaired auditory responses caused by cadmium in rats (Agirdir et al., 2002). To test whether zinc could likewise protect against cadmium-induced hair cell death in zebrafish, we co-treated fish with 30 μM of cadmium chloride and varying doses of zinc sulfate, based on doses that had previously been shown to protect against cadmium-induced defects in olfactory behavior in larval zebrafish (Heffern et al., 2018), with and without a 1-h zinc sulfate pretreatment. We found no significant reduction in hair cell death in response to cotreatment alone (Figure 4A), and only a slight reduction at the highest zinc sulfate dose tested 1,364 μM, which is equivalent to 88 μg/L zinc, in response to the 1-h pretreatment combined with cotreatment (Figure 4B). When treating fish with 1,364 μM zinc sulfate, 88 μg/L zinc, an hour before and in combination with a range of cadmium chloride doses, we failed to see significant protection (Figures 4C,D). Zinc has also been shown to kill hair cells on its own at a concentration of 0.5 mg/L in adult zebrafish (Montalbano et al., 2018), or to exacerbate hair cell death in response to other toxicants at a concentration 10 mM following round window application in guinea pigs (Nakagawa et al., 1997); however, we saw no evidence of this at the lower doses we used (Figure 4).

**Figure 5:**
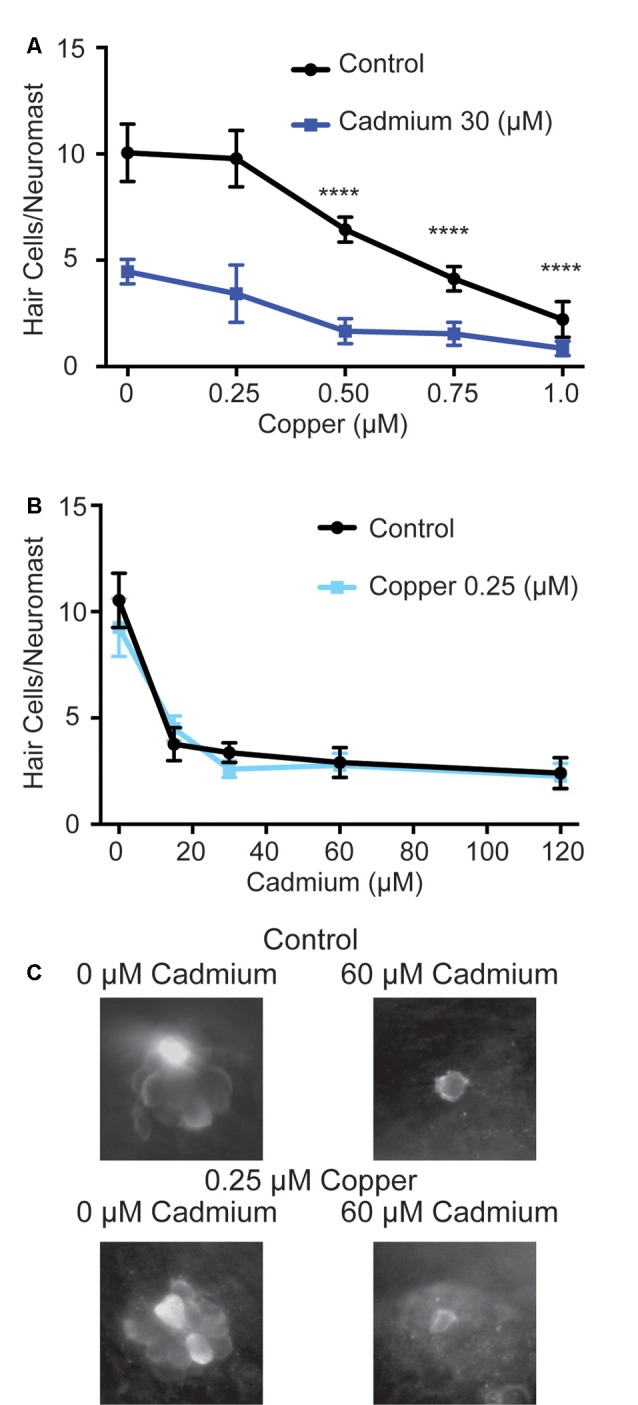
Copper fails to protect against cadmium-induced hair cell death. **(A)** At doses of 0.5 μM and higher copper sulfate caused significant hair cell death on its own. ****p < 0.0001 by Two-Way ANOVA and Šídák multiple comparisons test as compared to the 0-copper sulfate control. Using copper sulfate in concentrations up to 1 μM failed to protect against hair cell death in response to 30 μM of cadmium chloride. **(B)** The 0.25 μM dose of copper sulfate failed to show a significant reduction in hair cell death at all cadmium chloride doses tested. Data are displayed as mean ± standard deviation. *n* = 10 fish for all groups. **(C)** Representative images of the O2 Neuromast in control (top) and copper sulfate treated (bottom) fish following treatment with either 0 (left) or 60 (right) μM cadmium chloride.

While cadmium is not believed to travel through copper transporter family gene products, it has been shown that copper cotreatment can impair cadmium uptake in fish (Komjarova and Bury, 2014). Copper has also been shown to protect against hair cell death in response to the platinum-based chemotherapeutic cisplatin and the heavy metal lead in mammals (More et al., 2010; Liu et al., 2011), though it was unable to protect against cisplatin in fish (Thomas et al., 2013). To determine whether copper might protect hair cells in zebrafish from cadmium toxicity, we first tested various doses of copper sulfate to see if they could protect against 30 μM of cadmium chloride when fish were treated with copper sulfate for an hour before and while treated with cadmium chloride. Copper has previously been shown to be toxic to zebrafish hair cells on its own (Hernández et al., 2006; Linbo et al., 2006; Olivari et al., 2008; Mackenzie et al., 2012) and we likewise found all but the smallest dose of copper sulfate tested, 0.25 μM, caused significant hair cell death on its own while offering no protection against cadmium chloride (Figure 5A). We subsequently tested whether pre- and cotreatment with 0.25 μM copper sulfate would protect against a range of cadmium chloride doses but again failed to see any significant protection (Figures 5B,C).

## Discussion

Here, we show that cadmium can consistently cause hair cell death in the lateral line system of zebrafish larvae. This is consistent with prior work that has shown cadmium can cause hair cell death in fish (Faucher et al., 2006; Sonnack et al., 2015; Montalbano et al., 2018). Studies looking at hair cell death and/or hearing loss in response to cadmium exposure in mammals and humans, on the other hand, have had much less consistent results, with some studies showing that cadmium is damaging (Ozcaglar et al., 2001; Kim et al., 2008; Choi et al., 2012; Min et al., 2012; Liu et al., 2014; Choi and Park, 2017) and others showing no effect (Whitworth et al., 1999; Shiue, 2013; Klimpel et al., 2017; Carlson et al., 2018; Kang et al., 2018; Liu et al., 2018). This disparity might come from the fact that lateral line hair cells in fish are on the surface of the animal and therefore easily accessible to toxins. In contrast to this, for cadmium to reach hair cells in mammals it must first travel through the bloodstream where it is actively removed by the liver and kidneys (Swiergosz-Kowalewska, 2001; Yang and Shu, 2015; Satarug, 2018). This means presumably only a percentage of the cadmium an individual is exposed to will ever make it to the hair cells. The fact that some researchers did see either hair cell damage or hearing impairment in mammals following cadmium exposure suggests that it can cause damage in these systems as long as sufficient amounts are present. Therefore, understanding how this damage occurs is an important question to research further.

In this study, we attempted to elucidate how cadmium enters hair cells. We did this by blocking potential uptake mechanisms and looking for a decrease in cadmium-induced hair cell toxicity. We showed that blocking mechanotransduction activity either *via* a genetic mutation in *cdh23* mutants or pharmacologically *via* benzamil lead to a significant reduction in cadmium-induced hair cell death. While *cdh23* mutants still showed significant cadmium-induced hair cell death at higher doses of cadmium, benzamil appeared to show an almost complete elimination of hair cell death across the dose range tested. This is different from what was previously shown for copper, where amiloride could protect against only lower doses of copper (Olivari et al., 2008). The reason for the differences in resistance seen in *cdh23* mutants vs. benzamil treatment may be that unlike *cdh23*, benzamil is likely not blocking mechanotransduction selectively. Amiloride and benzamil are both considered nonselective epithelial sodium channel blockers and have been shown to be capable of blocking a number of channel types including some Ca^2+^, TRP, and K^+^ channels (Bielefeld et al., 1986; Tang et al., 1988; Dai et al., 2007; Castañeda et al., 2019). Therefore, while the mechanotransduction channel may serve as a major entry route for cadmium, cadmium could also be entering at a lower level through other channel types blocked by benzamil, thus allowing for the low level of hair cell death seen in *cdh23* mutants. The small remaining level of hair cell death seen in *cdh23* mutants in the absence of mechanotransduction activity is in contrast to other toxicants that kill hair cells in a mechanotransduction-dependent manner, such as cisplatin and aminoglycosides, where toxicity can be completely blocked (Wang and Steyger, 2009; Thomas et al., 2013).

To test other potential uptake routes for cadmium into hair cells, we next investigated whether zinc or copper could protect hair cells from cadmium-induced hair cell death potentially by competing for entry. We failed to see consistent protection from either of these ions; however, these experiments were complicated by the fact that both copper and zinc can kill hair cells on their own (Hernández et al., 2006; Linbo et al., 2006; Montalbano et al., 2018), requiring the use of lower concentrations of these ions. Copper, in particular, kills hair cells at doses considerably lower than cadmium. The zinc sulfate doses we used were based on previous work showing that 22 μg/L zinc could protect against cadmium-induced olfactory damage whereas lower and higher doses could not (Heffern et al., 2018). We failed to see protection at 22 μg/L of zinc which fish were exposed to as 336 nM zinc sulfate. We did see protection at the higher dose 88 μg/L zinc, which fish were exposed to as 1,346 μM zinc sulfate; however, that protection was inconsistent. Also, while benzamil was able to protect following the cotreatment with cadmium chloride, zinc sulfate required a 1-h pretreatment to show any effect.

While we failed to show consistent protection against cadmium-induced hair cell death from zinc, it has previously been shown that zinc can protect against cadmium-induced hearing loss in rats (Agirdir et al., 2002). Zinc can also protect against cadmium-induced nephrotoxicity (Liu et al., 1992; Tang et al., 1998). These experiments were looking over a longer period of cadmium treatment than we used. Potential mechanisms for this protection include the upregulation of metallothionein, prevention of gene expression changes caused by cadmium that normally lead to toxicity, and prevention of cadmium from displacing zinc off metal-binding enzymes (Andrews, 2000; Pinter and Stillman, 2015; Pan et al., 2017). Possibly these mechanisms do not have time to activate in the 3-h treatment window we are using. Also, we are exposing the fish to cadmium by adding cadmium chloride to the water they are swimming in. This is in contrast to mammalian studies that use more systemic treatments where cadmium is injected or given through drinking water. Presumably, given our short treatment times, only the apical part of hair cells, which are most accessible to the surrounding water, are exposed to cadmium. This may be the cause of the dominant role of mechanotransduction as a means of protecting against cadmium-induced hair cell death, as fewer other channels may be present on the apical area of the cell.

We also investigated whether two cilia-associated gene mutants that are resistant to aminoglycoside-induced hair cell death were similarly resistant to cadmium-induced hair cell death. Mutations in one of those genes, *ift88*, did lead to resistance in cadmium-induced hair cell death similar to what had previously been seen with aminoglycosides. Ift88 is a component of the IFT-B complex a group of proteins important for anterograde intraflagellar transport in cilia and also for cilia formation (Pazour et al., 2000; Cole, 2003). These mutants have previously been shown to have a decrease in FM1-43 uptake (Stawicki et al., 2016) and a decreased response to water jet stimulation (Kindt et al., 2012), suggesting impaired mechanotransduction activity though mechanotransduction is not eliminated as in *cdh23* mutants where no response is seen (Nicolson et al., 1998). Thus, *ift88* mutant’s resistance to cadmium-induced hair cell death further supports the role of mechanotransduction in this process. They are not as resistant as *cdh23* mutants, which fits the fact that mechanotransduction is not as impaired. We failed to see significant resistance to cadmium-induced hair cell death in mutants of the other gene tested, *cc2d2a*. Cc2d2a is part of a complex of proteins located at the base of the cilia known as the transition zone that is believed to function as gatekeepers for cilia (Garcia-Gonzalo et al., 2011; Chih et al., 2012). Unlike *ift88* mutants, these mutants do not show any impairment in FM1-43 uptake, suggesting normal mechanotransduction activity (Owens et al., 2008; Stawicki et al., 2016). This supports the hypothesis that the reduced cadmium-induced hair cell death seen in *ift88* mutants is due to the reduced mechanotransduction in these mutants rather than a general role of cilia in cadmium-induced hair cell death.

## Data Availability Statement

The datasets generated for this study are available on request to the corresponding author.

## Ethics Statement

The animal study was reviewed and approved by University of Washington IACUC, Lafayette College IACUC.

## Author Contributions

TS designed this study. Experiments were performed and data analyzed by TS, CS, IA, JB, and KT. The article was written by TS and CS. All authors contributed to manuscript revision and approved the submitted version.

## Conflict of Interest

The authors declare that the research was conducted in the absence of any commercial or financial relationships that could be construed as a potential conflict of interest.
